# The World Health Organization road map for neglected tropical diseases 2021–2030: implications for onchocerciasis elimination programs

**DOI:** 10.1186/s40249-021-00848-x

**Published:** 2021-05-17

**Authors:** Melissa Krizia Vieri, Makoy Yibi Logora, Kamran Rafiq, Robert Colebunders

**Affiliations:** 1 Global Health Institute, University of Antwerp, Kinsbergen Centrum, Doornstraat 331, 2610 Antwerp, Belgium; 2 Neglected Tropical Diseases Unit, Ministry of Health, Juba, Republic of South Sudan; 3 The International Society for Neglected Tropical Diseases, London, UK

**Keywords:** WHO road map, Onchocerciasis, Elimination, Epilepsy, Nodding syndrome, Community based program, Ivermectin

## Abstract

In its new roadmap for neglected tropical diseases, the World Health Organization proposes three important strategic shifts: (i) Stronger accountability which shifting from process to impact indicators; (ii) Intensified cross-cutting approaches; and (iii) Stronger country ownership. In this paper we discuss the implementation of these three strategies in the setting of a high onchocerciasis disease burden in South Sudan.

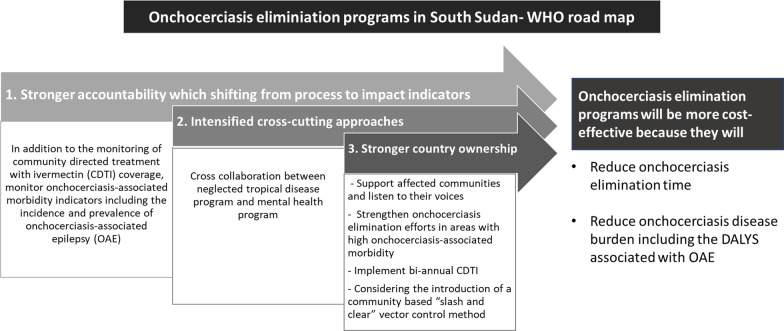

## Background

On January 28, 2021, the World Health Organization (WHO) formally launched its new road map for neglected tropical diseases (NTDs). During the virtual launch the WHO document ‘Ending the neglect to attain the Sustainable Development Goals: a road map for neglected tropical diseases 2021–2030’ [1] was presented. This document aims to strengthen the programmatic response to NTDs. It focuses on how cross-sectoral, integrated interventions, smart investment and community engagement can strengthen and sustain health systems [1]. Three important strategic shifts are proposed: (i) Stronger accountability—shifting from process to impact indicators and accelerating programmatic action, (ii) Intensified cross-cutting approaches, (iii) Stronger country ownership, improved roles of stakeholders, clearer roles and responsibilities to deliver on 2030 targets [1].

In this commentary we discuss the value of these three important shifts for the onchocerciasis (river blindness) elimination program in South Sudan.

## Main text

The onchocerciasis-endemicity in South Sudan is amongst the highest in Africa, with the disease being prevalent in around half of the country and with more than 7.5 million people at risk [2]. Civil war and insecurity have led to interruption of the community directed treatment with ivermectin (CDTI) program. The most highly endemic foci of onchocerciasis in South Sudan are in Western Equatoria and Northern and Western Bahr el Ghazal regions [3]. However, in 2006 only 26% of the eligible population received ivermectin [3]. This has resulted in high onchocerciasis transmission and a major public health crisis with a high prevalence of onchocerciasis-associated morbidities including skin and eye diseases but also epilepsy [4–6]. High numbers of persons with nodding syndrome and other forms of epilepsy have been documented in the Western Equatoria region of South Sudan [4–6], but based on rumors most likely high numbers of persons with epilepsy will also be found in other onchocerciasis-endemic regions. So far, only a limited number of population-based surveys to determine the prevalence of onchocerciasis-associated morbidity have been performed in South Sudan, and only in Western Equatoria. In 2013, during a small household survey in Mvolo, one in six children was found to have epilepsy, and one in two households had at least one child with epilepsy [6]. In 2018, in villages from Maridi County, in a door-to-door study, an epilepsy prevalence of 4.4% was documented with a prevalence of 11.9% in a village close to the Maridi dam, the blackfly (the vector transmitting onchocerciasis) breeding site in the area [5]. In this area, 85.2% of persons with epilepsy met the criteria of onchocerciasis-associated epilepsy and 45.5% the criteria of nodding syndrome [7]. The incidence of epilepsy was estimated to 373.9 per 100 000 persons [5]. This high epilepsy prevalence and incidence was considered to be the consequence of the weak onchocerciasis elimination program in South Sudan. Indeed, only 7209 (40.8%) of the population who participated in the door-to-door survey in 2018 reported to have taken ivermectin in 2017 [5].

## How to tackle this situation?

1. Stronger accountability: Currently, onchocerciasis elimination programs are mainly monitoring the process of the program. Their main indicator of performance is CDTI coverage which is mainly monitored by reviewing the reports of the community drug distributors (CDDs) of ivermectin. However, CDTI coverage data provided by CDDs are not reliable. Indeed, some CDDs may not be completely honest in their reporting. They may know how many tablets they distributed but they may not know whether these tablets were taken. In addition, they may not include in their reports persons of households that were not visited. Therefore, CDTI coverage calculations based on these reports may use denominators that underestimate the number of eligible persons in the area. As a consequence, CDTI coverage data calculated based on CDD reports generally overestimate the real CDTI coverage. Therefore, it is advised to carry out coverage surveys by an independent research team. However, such surveys are infrequently done and moreover rely on self-reported ivermectin intake by the population. Social desirability may influence the way people respond during interviews and therefore such surveys also may overestimate the CDTI coverage.

In many onchocerciasis endemic areas, annually and certainly biannually distribution of ivermectin has been shown to be effective in reducing onchocerciasis-associated morbidity and in eliminating onchocerciasis as a public health problem [2, 8]. This explains the decreased emphasis on monitoring changes in morbidity as response to treatment. However, recent epidemiological studies have shown that in certain onchocerciasis-endemic areas in Africa, where CDTI coverage has been sub-optimal or interrupted, there remains a high onchocerciasis disease burden [5, 9, 10].

Therefore, it is important that in onchocerciasis endemic areas, onchocerciasis-associated morbidity such as itching, skin lesions, blindness and epilepsy are being monitored. A high prevalence of epilepsy in an onchocerciasis-endemic area suggests that this area may be a hotspot for onchocerciasis transmission and that the CDTI coverage in this area is sub-optimal. Certainly, a high incidence of new onset epilepsy in children and youngsters between the ages of 3 and 18 years, in an onchocerciasis-endemic area should be a reason to assess the performance of the CDTI program. In onchocerciasis-endemic areas in South Sudan, where there are no pigs, and therefore no neurocysticercosis, most epilepsy without an obvious cause, starting in previously healthy children 3 and 18 years should be considered as onchocerciasis-associated epilepsy [7]. Therefore, in these areas, epilepsy incidence together with itching and blindness should be used as an impact indicator of the onchocerciasis elimination program.

2. Cross-cutting approaches: Using this epilepsy-based proxy-measure of onchocerciasis prevalence will need partnerships outside of the traditional mass drug administration and supply chain partnerships. Adopting this proxy-measure implies a close collaboration between the NTD program and the health care system providing anti-seizure treatment for persons with epilepsy. NTDs are increasingly recognized as major drivers of psychosocial morbidity in affected individuals and their caregivers [11]. However, so far mental health problems caused by onchocerciasis-associated epilepsy have not been internationally recognized. Given the strong epidemiological evidence for the association between onchocerciasis and epilepsy, onchocerciasis-associated epilepsy should be considered as one of the clinical presentations of onchocerciasis [12]. For those who still do not want to recognize this, onchocerciasis-associated epilepsy should be considered as new NTD because it only occurs in tropical areas and is a neglected public health problem. A collaboration of the onchocerciasis elimination program with the mental health program has many advantages. Epilepsy surveillance may identify onchocerciasis “hotspots” and in areas with a weak/interrupted CDTI program a high prevalence of epilepsy is to be expected. Increasing the awareness about onchocerciasis-associated epilepsy among public health decision makers, health care workers and affected communities will increase ivermectin intake and decrease epilepsy related stigma and discrimination.

3. Country ownership: Currently, onchocerciasis elimination programs are focusing on onchocerciasis elimination mapping instead of identifying areas/communities of high onchocerciasis-associated morbidity and prioritizing strengthening onchocerciasis elimination programs. In Uganda, bi-annual ivermectin distribution and larvicide stopped nodding syndrome epidemic [13]. To observe a successful outcome as observed in Uganda, these communities need an additional amount of ivermectin and resources to organize bi-annual CDTI. So far, the South Sudanese NTD program has been unable to obtain bi-annual CDTI. Moreover, the last CDTI round in South Sudan was in November 2020 and a new round, because of the COVID-19 pandemic, is only being organized in February 2021. To tackle the high onchocerciasis disease burden in Maridi, in November 2019, the local community of Maridi, together with the national NTD program, initiated a community based “slash and clear” vector control intervention at the Maridi dam [14]. This community based “slash and clear” method was found to be effective in reducing blackfly biting rates and its effect on onchocerciasis transmission is now being evaluated. Onchocerciasis elimination programs could be more cost-beneficial if areas with high onchocerciasis-associated morbidity are prioritized. Indeed, by decreasing onchocerciasis-associated morbidity it will also decrease the important psycho-socio and economic consequences of these morbidities. In South Sudan, at the national level, but also county- and community level, epilepsy including nodding syndrome is recognized as a major public health problem in onchocerciasis-endemic areas. Initiatives have been taken to increase the awareness about onchocerciasis-associated epilepsy and to stress the importance of high CDTI coverage to prevent this form of epilepsy. In addition, in the Maridi and Mundri area in the Western Equatorial region, a community-based program has started to decrease the epilepsy treatment gap. However, at the international level the burden of disease caused by epilepsy in onchocerciasis-endemic regions is still not recognized as a problem that urgently needs to be addressed. Persons with epilepsy particularly in onchocerciasis-endemic areas are stigmatized and discriminated. In those areas, epilepsy often occurs clustered in certain households and villages, and therefore families are often considered as cursed [15]. Moreover, because of the lack of anti-seizure medication, persons with epilepsy often present severe intellectual disabilities. Therefore, if affected communities are not supported, their voices will not be heard. Experience with other potentially stigmatizing condition such as HIV infection has shown the power of involving affected communities to influence health policies. Currently, in onchocerciasis elimination programs eliminating the parasite seems to be the main focus of international interest and not the person or community affected by onchocerciasis. Supporting onchocerciasis affected communities and listening to their voices is essential for developing more effective elimination programs.

## Conclusions

The three strategic shifts proposed by WHO will be useful for the onchocerciasis elimination program in South Sudan. In addition, if implemented with a focus on affected communities, they will be important to reduce the suffering of affected populations.

## Data Availability

Not applicable.

## Abbreviations

CDDs: Community drug distributors
CDTI: Community directed treatment with ivermectin
NTDs: Neglected tropical diseases
WHO: World Health Organization
